# Diffuse Alveolar Hemorrhage Due to Cannabis Inhalation

**DOI:** 10.7759/cureus.76755

**Published:** 2025-01-01

**Authors:** Maria Jaquez Duran, Sindhaghatta Venkatram, Gilda Diaz-Fuentes

**Affiliations:** 1 Pulmonary and Critical Care, BronxCare Health System, Bronx, USA

**Keywords:** cannabis use, cannabis use disorder, diffuse alveolar hemorrhage, diffuse lung injury, diffuse pulmonary hemorrhage

## Abstract

We report the case of a 34-year-old female who presented with acute respiratory failure and hemoptysis, requiring intubation and mechanical ventilation. Fiberoptic bronchoscopy revealed progressively bloodier returns during lavage, consistent with diffuse alveolar hemorrhage (DAH). This case highlights DAH as a rare but severe complication associated with cannabis use. DAH involves bleeding into the alveolar spaces, impairing gas exchange and causing respiratory distress. While cannabis use is not a well-established cause, this case underscores its potential to trigger serious respiratory complications.

Recurrent DAH episodes can lead to long-term complications, such as interstitial fibrosis and emphysema, resulting in obstructive spirometry patterns. Acute complications, including shock, renal failure, and barotrauma, further highlight the critical need for early intervention. Raising awareness among healthcare providers about the potential link between cannabis use and DAH is essential for prompt recognition and management, ultimately improving patient outcomes. This case adds to the limited literature on cannabis-induced DAH, emphasizing its clinical significance.

## Introduction

The increasing prevalence of cannabis use, both recreationally and medically, has prompted growing concern over its long-term effects on lung health. Cannabis (also referred to as marijuana, "pot," or "weed") is now recognized as the second most smoked substance after tobacco. It is the most widely used recreational drug in the world, and its smoke contains numerous harmful chemicals, including tar, nitrosamines, hydrocyanic acid, ammonia, and a variety of carcinogens. These substances are known to adversely impact respiratory health, mirroring many of the risks associated with tobacco smoking [1].

One of the more severe and less commonly discussed complications linked to cannabis use is diffuse alveolar hemorrhage (DAH). This rare but serious condition involves bleeding into the alveolar spaces, impairing gas exchange and leading to acute respiratory distress. The smoking patterns typical of cannabis users - characterized by deep inhalation and prolonged breath-holding - may exacerbate the deposition of these harmful chemicals, increasing the risk of significant lung injury [2].

In this report, we present the case of a 34-year-old female who was hospitalized with rapid onset of dyspnea, hemoptysis, and acute respiratory failure (ARF) following the combined use of cannabis and tobacco. Her subsequent diagnosis of DAH highlights the critical need for awareness of the respiratory risks associated with cannabis smoking.

## Case presentation

A 34-year-old female with no significant past medical history presented to the intensive care unit with a one-day history of hemoptysis and ARF. The patient was an active smoker of both tobacco and cannabis but denied vaping or using bongs. She reported a productive cough with progressively worsening blood-streaked sputum. She denied any systemic symptoms, such as fever, weight loss, or recent respiratory infections.

**Figure 1:**
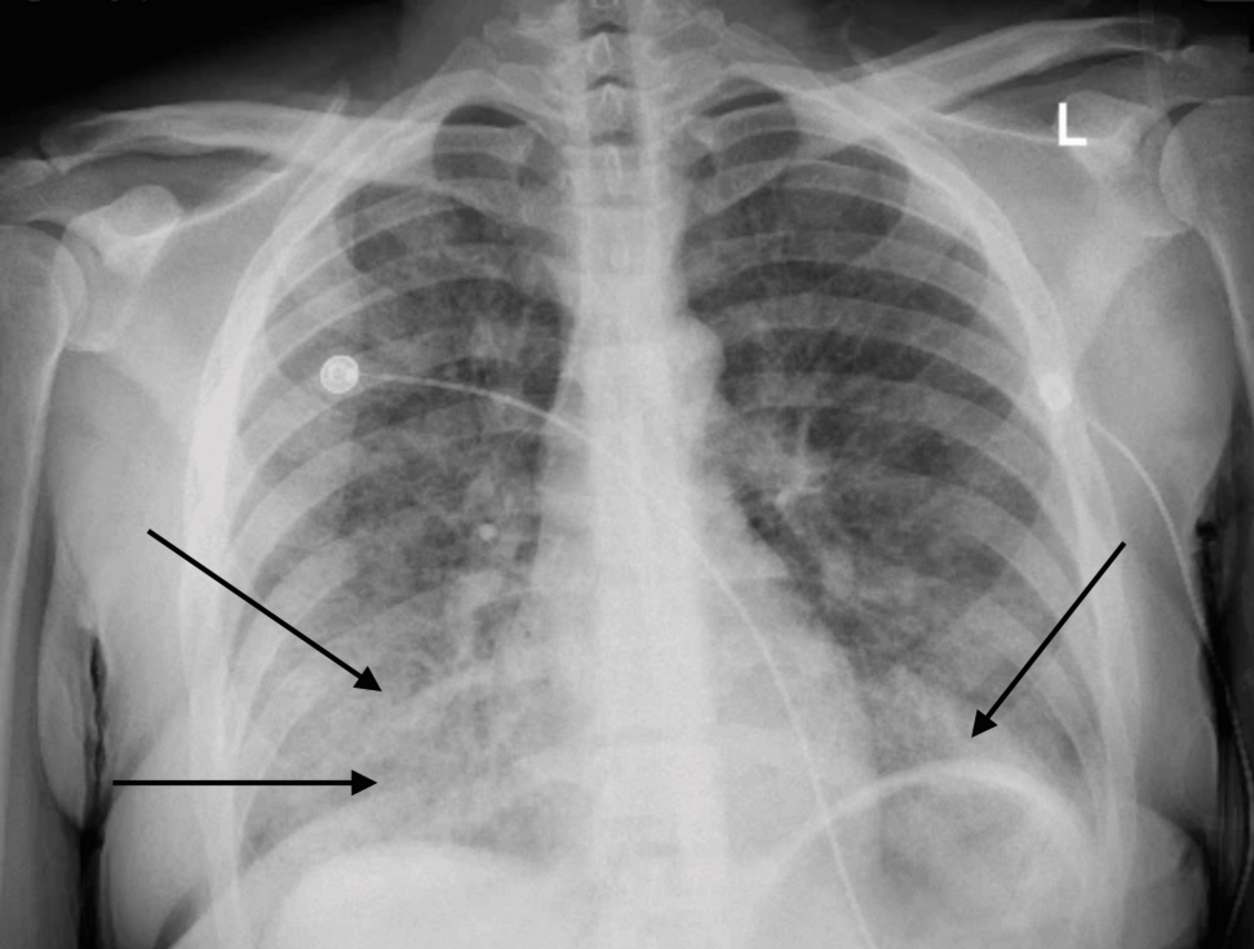
Initial chest X-ray. Black arrows showing bilateral lower lungs infiltrates worse on the right

**Figure 2:**
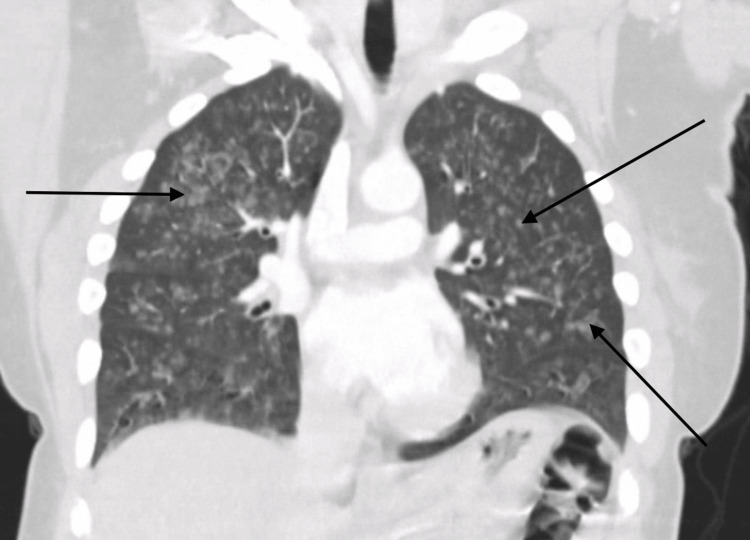
Computed tomography of the chest. Black arrows indicating diffuse ground-glass opacities.

On initial evaluation, the patient was alert and oriented but noted to be hypoxic with oxygen saturation of 91% on room air and required supplemental oxygen. Chest X-ray initially revealed bilateral patchy opacities (Figure 1), and contrast-enhanced computed tomography (CT) of the chest revealed diffuse ground-glass opacities (GGOs) (Figure 2). Laboratory tests showed an initial hemoglobin level of 13.3 g/dL, which precipitously dropped to 9.3 g/dL (reference range: 12.0-16.0 g/dL) within 24 hours. Urine toxicology was positive for cannabis, while renal function and coagulation studies were unremarkable.

**Figure 3:**
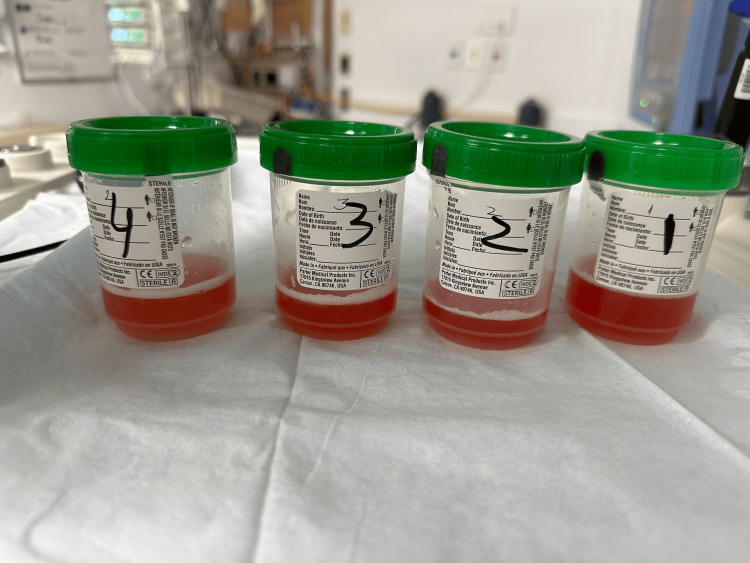
Bronchoalveolar lavage obtain during bronchoscopy Serial bloody aliquots

Her condition deteriorated rapidly, with massive hemoptysis necessitating intubation and mechanical ventilation. The patient was managed with lung-protective ventilation strategies and underwent an emergency fiberoptic bronchoscopy (FOB). Bronchoscopy findings were notable for the absence of endobronchial lesions but demonstrated progressively bloodier returns during sequential lavage, consistent with DAH (Figure 3).

A broad differential diagnosis was considered and was classified as either autoimmune or non-autoimmune, including infections, coagulation problems, cardiovascular illnesses, cancer, and illegal drug use. A broad differential diagnosis was considered and was classified as either autoimmune or non-autoimmune, including infections, coagulation problems, cardiovascular illnesses, cancer, and illegal drug use. Comprehensive infectious and autoimmune workup including blood and sputum bacterial cultures, viral and fungal cultures, acid-fast bacilli culture, and collagen vascular profile was negative.

The patient was empirically started on broad-spectrum antibiotics with ceftriaxone, vancomycin, and doxycycline, and pulse-dose corticosteroids.

Her clinical course was complicated by cardiac arrest, likely secondary to hypoxia. She was successfully resuscitated and continued on supportive therapy, including mechanical ventilation and hemodynamic stabilization. Following stabilization, the patient showed gradual improvement. She was extubated and weaned off supplemental oxygen. After 10 days of hospitalization, she was discharged home in stable condition.

Follow-up and outcome

**Figure 4:**
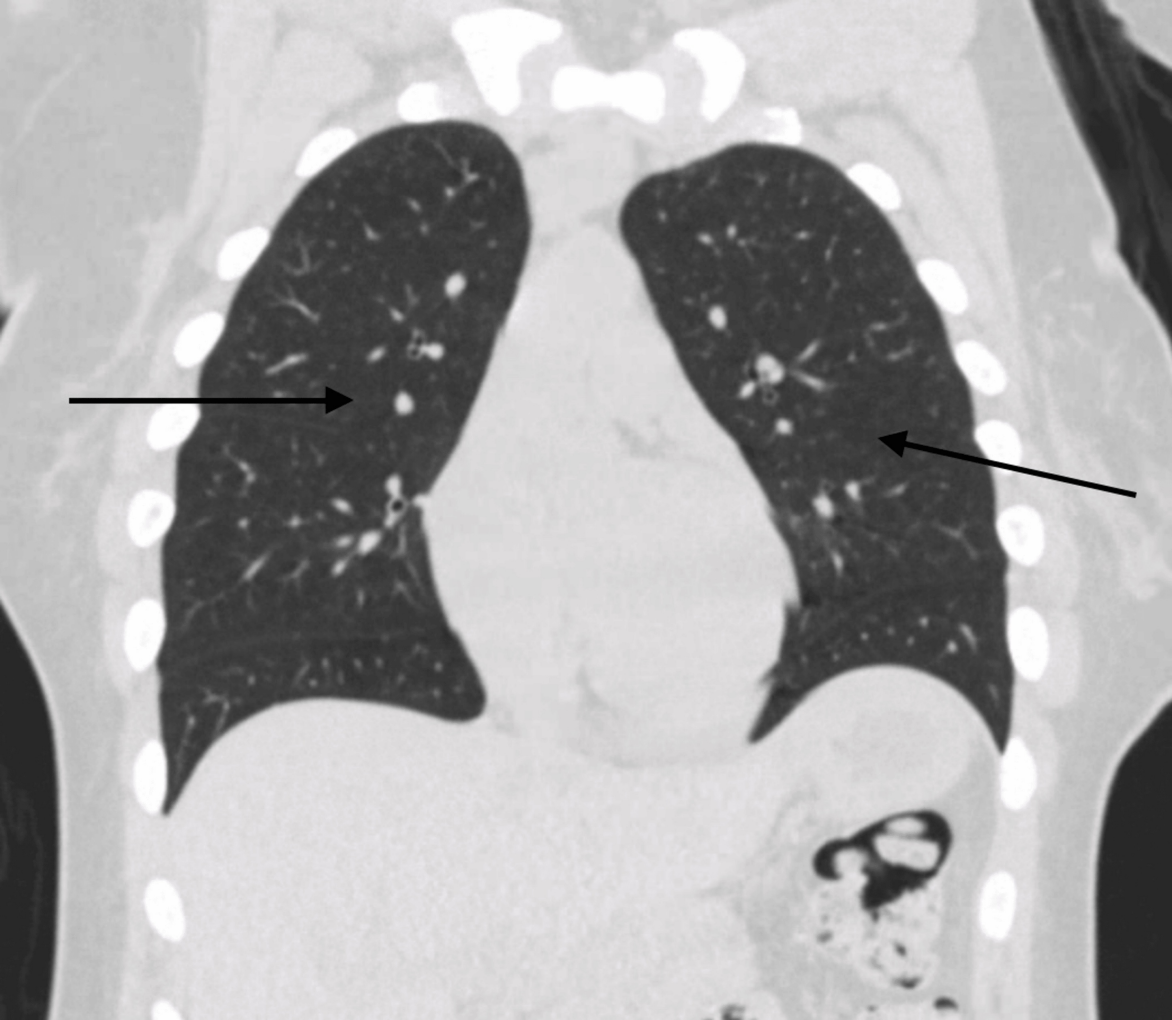
Repeat computed tomography of the chest two months later. Black arrows showing complete resolution of ground-glass opacities

At a follow-up visit in the pulmonary clinic, the patient reported cessation of cannabis use, with no further episodes of hemoptysis or respiratory symptoms. Repeat CT of the chest two months after discharge showed complete resolution of GGOs (Figure 4).

This case highlights the potential for serious respiratory complications, including DAH, associated with cannabis smoking, and underscores the importance of early diagnosis and aggressive management.

## Discussion

Cannabis use has become increasingly prevalent in recent years, as evidenced by data from the 2021 National Survey on Drug Use and Health, which reported that approximately 49.6 million people used cannabis that year. This represents a steady rise in use compared to previous years. Among adults aged 18-25 years, the prevalence was even higher, at 34%, underscoring a particularly strong trend of increased consumption among younger adults [1,3].

Cannabis is now the second most commonly inhaled substance after tobacco. Data from 2011 further highlights this trend, with 36.4% of high school seniors reporting cannabis use, reflecting its widespread acceptance among adolescents and young adults. The primary methods of consumption include smoking cannabis cigarettes (joints), vaping using electronic cigarettes, and inhalation via water pipes (bongs).

These routes of inhalation expose the lungs to harmful chemicals, including tar, carbon monoxide, and various carcinogens, which can cause significant respiratory damage. Patterns of deep inhalation and prolonged breath-holding unique to cannabis use may enhance the deposition of these toxic substances, increasing the risk of conditions such as DAH, as illustrated in this case.

This growing trend of cannabis use, especially through inhalation, highlights the need for further research into its respiratory effects and increased awareness of potential complications.

Respiratory complications of inhaled cannabis

Inhaled cannabis has been linked to a variety of respiratory complications, including barotraumas such as pneumothorax, pneumomediastinum, and subcutaneous emphysema. Other complications include chronic bronchitis cellular alveolitis and DAH.

Cannabis smoking also increases the lungs' susceptibility to infections by inhibiting nitric oxide synthase, thereby impairing the production of reactive nitrogen species, which are crucial for bacterial killing. In vitro animal studies have further demonstrated that tetrahydrocannabinol (THC), the main psychoactive component of cannabis, binds to leukocytes and produces an immunosuppressive response by modulating T-cells, natural killer cells, and macrophages. This weakened immune response is associated with an increased risk of bacterial and fungal infections, as noted by Han et al. [2].

Cannabis and chronic respiratory effects

While cannabis smoking is associated with bronchial inflammation and hypersecretion, studies suggest that it also increases forced vital capacity (FVC), unlike tobacco smoking. The relationship between cannabis use and chronic respiratory conditions such as bronchitis, chronic obstructive pulmonary disease (COPD), and lung cancer often involves confounding factors, particularly concurrent tobacco use.

DAH and cannabis use

DAH is a rare complication despite the widespread use of cannabis. Alternate theories to explain DAH among cannabis users involve methods of inhalation, particularly through bongs or vaping.

Bong use: DAH has been attributed to the inhalation of toxic acid anhydrides released when plastic bottles are heated during bong use.

Vaping: The role of propylene glycol, a common solvent in vaping liquids, has been implicated in developing DAH. However, its low toxicity as per studies such as those by Wieslander et al. and Philips et al. makes this association inconclusive [4,5].

Despite these theories, it is important to note that DAH has been reported in cannabis users who did not use bongs or vape. Such cases, including our patient, highlight the rarity and complexity of DAH and suggest other unidentified mechanisms of injury related to cannabis smoke inhalation.

Cannabis-induced DAH is a rare clinical entity, and its exact prevalence remains poorly established. While several cases have been reported in the literature, many involve the co-use of other substances, complicating the attribution of DAH solely to cannabis. However, DAH is observed more frequently in individuals with underlying infectious processes, autoimmune disorders, such as lupus or systemic vasculitides, certain medications, including anticoagulants and chemotherapeutic agents, and exposure to inhaled toxic substances [6].

The clinical presentation of DAH is nonspecific and varies widely, often depending on the severity of the condition. Common features include hemoptysis (may be absent in some cases), dyspnea, cough, and chest pain. Imaging may reveal bilateral lung infiltrates. The clinical course can range from mild respiratory symptoms to life-threatening ARF, as seen in this case. Laboratory findings are typically nonspecific. Common findings include anemia, secondary to blood loss and elevated inflammatory markers. While imaging and FOB are instrumental in diagnosing DAH, establishing the etiology often requires exclusion of infectious, autoimmune, and other contributing factors [7].

The pathophysiologic mechanism of cannabis-induced DAH is not well understood but is believed to be characterized by bleeding into alveoli secondary to the disruption of the alveolar-capillary basement membrane as a result of an injury at the level of alveolar microcirculation. Acute irritation of the lung parenchyma by cannabis smoke, which contains numerous toxic and inflammatory agents, may play a critical role. Chronic airway inflammation caused by repeated exposure to cannabis smoke can lead to conditions such as chronic bronchitis and airway remodeling. These changes may compromise the structural integrity of the lungs, reducing their elasticity and making them more susceptible to injury.

Additionally, cannabis use has been associated with an increased risk of pulmonary infections due to its immunomodulatory effects, including the suppression of immune cell activity. This weakened immune response may contribute to the development of inflammatory processes in the lung that predispose to hemorrhage. Together, these mechanisms - irritation, inflammation, structural changes, and infection susceptibility - likely act synergistically to increase the risk of DAH in cannabis users.

The diagnosis of DAH is based on a clinical constellation of characteristic features and confirmation through bronchoscopic findings. Sequential bronchoalveolar lavage (BAL) of the affected lung segments is a key diagnostic tool, revealing hemorrhagic fluid that persists without clearing after sequential aliquots, which confirms the diagnosis. This approach not only identifies DAH but also helps differentiate it from other causes of pulmonary infiltrates or bleeding [8].

FOB plays a crucial role in the diagnostic process by helping localize the potential source of bleeding, exclude infections or malignancies, and identify any structural abnormalities in the airways. Analysis of BAL fluid may reveal hemosiderin-laden macrophages, a hallmark finding in DAH. However, these cells typically take up to 72 hours to accumulate. The Golde score is often used to quantify hemosiderin content in macrophages, with a score greater than 20% being strongly indicative of DAH [9].

Histologically, DAH is associated with various patterns, including pulmonary capillaritis, bland pulmonary hemorrhage, diffuse alveolar damage, and other miscellaneous histologies. Pulmonary capillaritis is the most common histopathologic finding in DAH and is characterized by inflammation and damage to the alveolar capillaries, which contributes to the hemorrhagic process. This combination of clinical, bronchoscopic, and histologic findings is essential for the accurate diagnosis and management of DAH [10].

Radiological findings

Typical pulmonary radiological findings of DAH include diffuse bilateral alveolar infiltrates, which are often described as GGOs on chest CT scans or consolidations on chest X-rays. However, in the early stages, chest X-rays can fail to capture the full extent of the disease, and serial X-rays or chest CT should be considered for a prompt and accurate diagnosis. The infiltrates observed in DAH are typically patchy or confluent and can progress rapidly, reflecting the alveolar spaces filling with blood. This pattern is usually non-segmental and predominantly affects the dependent lung regions [10].

Chest CT is more sensitive than chest X-rays in detecting the features of DAH. GGOs, as seen on CT scans, are more prominent and provide a clearer delineation of the extent of alveolar hemorrhage compared to consolidations. Unlike infectious pneumonia, the infiltrates in DAH generally lack air bronchograms, which can aid in distinguishing between these conditions. In the acute setting, chest imaging may appear normal in up to 50% of cases, which highlights that normal radiological findings do not rule out the diagnosis. Rapid imaging assessment combined with clinical evaluation is crucial for timely identification and intervention in cases of DAH [11].

Mortality

The overall mortality of DAH from all causes ranges from 20% to 40%, influenced by the underlying etiology, timeliness of diagnosis, and effectiveness of management strategies. In a case series by Alexandre et al., the reported all-cause mortality was 21%, while de Prost et al. reported an in-hospital mortality of 25% and a mortality rate of 16% during the follow-up period after discharge. Patients with an autoimmune etiology for DAH tend to have a poorer prognosis, as they are less responsive to treatment and experience higher mortality rates [12].

Recurrent episodes of DAH are linked to long-term complications, including interstitial fibrosis and emphysema, which can result in the development of obstructive patterns on spirometry. In the acute setting, DAH is often associated with severe complications such as shock, renal failure necessitating hemodialysis, and barotrauma. These complications highlight the critical nature of timely intervention in preventing morbidity and mortality [12].

Currently, there are insufficient data to definitively establish the mortality rate of DAH specifically caused by cannabis use. Further research is necessary to better understand the prognosis and potential long-term implications of cannabis-induced DAH.

## Conclusions

Cannabis use is highly prevalent in the U.S. population and continues to increase across all age groups, especially in states where its use has been legalized. While cannabis is often perceived as relatively safe, patients and healthcare providers must be aware of the rare but potentially life-threatening pulmonary complications associated with its use, such as DAH. Public health awareness is essential, as cannabis use continues to rise, to inform individuals of the potential risks and encourage early recognition of symptoms. DAH, though rare, is a rapidly progressive and potentially fatal condition. Delayed diagnosis and treatment can significantly increase mortality, particularly if healthcare providers do not associate cannabis use with the development of DAH. Raising awareness among healthcare providers about the connection between cannabis use and DAH is critical to ensure early detection, timely intervention, and better outcomes. As cannabis use becomes more widespread, research into its health impacts, including rare life-threatening complications such as DAH, is imperative for informing both clinical practice and public health strategies.
